# A cross-curricular physical activity intervention to combat cardiovascular disease risk factors in 11-14 year olds: 'Activity Knowledge Circuit'

**DOI:** 10.1186/1471-2458-9-466

**Published:** 2009-12-15

**Authors:** Gareth Knox, Julien S Baker, Bruce Davies, Susan Faulkner, Jaynie Rance, Anwen Rees, Kelly Morgan, Non Thomas

**Affiliations:** 1 Cardiff School of Sport, University of Wales Institute Cardiff, Cardiff, UK; 2 Health and Exercise Sciences, School of Sciences, University of the West of Scotland, UK; 3 Faculty of Health, Sport and Science, University of Glamorgan, Pontypridd, UK; 4 Department of Psychology, Careers and Education, University of Glamorgan, Pontypridd, UK; 5 School of Health Sciences, Swansea University, Swansea, UK; 6 School of Human Sciences, Swansea University, Swansea, UK

## Abstract

**Background:**

Cardiovascular disease is the leading cause of mortality worldwide. Risk factors associated with cardiovascular disease have been shown to track from childhood through to adulthood. Previous school-based physical activity interventions have demonstrated modest improvements to cardiovascular disease risk factors by implementing extra-curricular activities or improving current physical education curriculum. Few have attempted to increase physical activity in class-room taught curriculum subjects. This study will outline a school-based cross-curricular physical activity intervention to combat cardiovascular disease risk factors in 11-14 year old children.

**Method/Design:**

A South Wales Valley school of low socio-economic status has been selected to take part. Participants from year eight (12-13 years) are to be assigned to an intervention group, with maturation-matched participants from years seven (11-12 years) and nine (13-14 years) assigned to a control group. A cross-curricular physical activity intervention will be implemented to increase activity by two hours a week for 18 weeks. Participants will briskly walk 3200 m twice weekly during curriculum lessons (60 minutes duration). With the exception of physical education, all curriculum subjects will participate, with each subject delivering four intervention lessons. The intervention will be performed outdoors and on school premises. An indoor course of equal distance will be used during adverse weather conditions. Cardiovascular disease risk factors will be measured pre- and post-intervention for intervention and control groups. These will take place during physical education lessons and will include measures of stature, mass, waist, hip, and neck circumferences, together with skinfold measure's taken at four sites. Blood pressure will be measured, and fitness status assessed via the 20 m multi-stage fitness test. Questionnaires will be used to determine activity behaviour (physical activity questionnaire for adolescence), diet (seven day food diary) and maturation status. Fasting blood variables will include total cholesterol, low-density lipoprotein cholesterol, high density lipoprotein cholesterol, triglycerides, insulin, glucose, high-sensitivity C-reactive protein, interleukin-6, adiponectin, and fibrinogen. Motivational variables and psychological well-being will be assessed by questionnaire.

**Discussion:**

Our study may prove to be a cost effective strategy to increase school time physical activity to combat cardiovascular disease risk factors in children.

**Trial Registration:**

[NCT00998478]

## Background

Cardiovascular disease (CVD) is the leading cause of mortality worldwide [1]. In 2005 there was a reported 17.5 million deaths related to the disease, representing 30% of the global population [2]. Risk factors for CVD including obesity, blood pressure, blood lipids and lipoproteins have shown to track from childhood through to adulthood [3,4]. Metabolic syndrome (MetS) is the co-existence of multiple risk factors including hyperinsulinaemia, glucose intolerance, hypertension, decreased levels of high-density lipoprotein cholesterol (HDL-C) and elevated triglycerides (TG) [5]. At least one MetS risk factor was evident in one third of children (12-19 years) from the Third National Health and Nutritional Survey [6]; whereas one in ten was diagnosed with the syndrome itself when using the National Cholesterol Education Programme - Third Adult Treatment Panel definition. The prevalence of overweight and obesity for children (4-18 years) in the UK is reported to be 15 and 4% respectively [7]. Furthermore, in comparison to England (2.9%) obesity was more prevalent for those living in Scotland (7.6%) and Wales (6.5%).

For many, overweight and obesity is the result of the combined effect of excess energy consumption and inadequate physical activity (PA) [8]. Previously, for children and adolescents PA has correlated negatively with body fatness and insulin resistance [9-11]. Conclusions regarding associations with blood lipids and lipoproteins are less clear. Although a recent meta-analysis reported no effect of aerobic exercise on non-high-density lipoprotein cholesterol, further studies are warranted [12]. Despite this, significant inverse relationships between PA and metabolic risk scores have recently been reported [13,14], highlighting the importance of PA on childhood health. Schools are thought to be the ideal setting to implement PA interventions to combat CVD risk factors, with access to large populations from a variety of social classes, coinciding with a monitoring structure already in place [15,16].

The association between psychosocial variables and obesity is complex. Research findings in this area indicate that there is a relationship between depression in youth and subsequent obesity [17,18]. The stigma attached to being overweight may affect peer relationships and may result in children and adolescents feeling less able to participate in PA and more likely to suffer from psychological distress [19,20].

The impact of PA on psychological health and well being has been noted for some years. Biddle et al. [21] reported that PA was associated with reduced levels of anxiety and depression and improved self-esteem, mood and cognitive function. In addition, epidemiological evidence suggests that PA is associated with a decreased risk of developing depression [22]. Exercise programmes have consistently been found to be as effective in reducing levels of depression in adult samples as a variety of standard psychotherapeutic treatments [23].

The relationship between PA and psychological well-being needs further investigation. Physical activity can have a positive effect on self-esteem and physically active children tend to suffer from fewer mental health problems [24]. In a review of PA in children and adolescents, Biddle et al. [25] suggest that a lack of PA in children has been associated with a number of psychosocial variables and perceived barriers. Positive effects on self-esteem are evident for all people participating in PA however the impact on self-perceptions is particularly strong for children [26]. A different pattern of psychosocial variables seems to be important for adolescents including perceived competence, and achievement orientation.

Previous school-based interventions targeting both children and adolescents have demonstrated modest improvements to CVD risk factors, including obesity, insulin resistance, blood pressure, and blood lipids [15,27-29]. Many of these school-based interventions have also looked to improve diet. Most school-based PA interventions have looked to improve physical education (PE) lessons or to provide extra-curricular activities [15,30,31]; few studies have sought to increase PA levels by integrating exercise into what would normally be classroom delivered subjects.

Recently an intervention by Reed et al. [32] used a whole school approach targeting six action zones to increase elementary school children's (9-11 years) PA. The only prescriptive component was to introduce three 15-minute periods per week of classroom PA. This allowed participants to carry out activities including skipping, dancing and resistance exercises. Sixteen months into the programme, improvements in aerobic fitness and blood pressure were evident, but no effect was observed for blood lipids, apolipoprotein B, C-reactive protein, and fibrinogen. Despite detail pertaining to the duration of time provided for participants to perform PA, there was no information regarding the amount of actual PA performed. It is evident that more population-based prevention programs are needed [33]. Targeting class-room based curriculum subjects with an increase in PA, may display favourable outcomes in child CVD risk status.

## Method/Design

### Objectives

This study aims to implement an 18-week school-based cross-curricular exercise intervention to improve CVD risk status, psychological well-being, and motivation to exercise in 12 to 14 year old children.

### Primary Objectives

1. Reduce measures of obesity including body mass index (BMI), waist, hip, and neck circumferences, and total skinfold thickness.

2. Reduce systolic and diastolic blood pressure.

3. Improve aerobic fitness profiles and increase PA behaviour.

4. Improve blood lipid profiles and lower levels of plasma inflammatory markers.

5. To assess the impact of motivation to exercise on physiological and psychological outcomes following the cross-curricular intervention

6. To influence psychological well being (mood and negative affect), self concept, and cognitive performance in an intervention group compared with a control.

### Secondary Objectives

Determine associations between CVD risk factors measured at baseline in 11-14 year old children and adolescents. To investigate the relationships between specific physiological and psychological changes between and within participants.

### Participants

A South Wales Valley school of low socio-economic status (SES) has been selected to take part. Free school meal eligibility was used to ascertain a valid measure of SES [34]. Of all 11-16 year olds attending the school, a reported 28% are eligible for free school meals which is above the unitary authority average of 25% [35]. Following an explanation of the study's aim to the school's headmaster, gatekeeper consent has been granted for participants from years seven (11-12 years), eight (12-13 years), and nine (13-14 years) to take part. Participants from year eight will be assigned to an exercise intervention; maturation matched participants from years seven and nine will form a control group. A member of the research team will outline the study to all children from years seven, eight, and nine during the relevant school assemblies. This will be followed by the distribution of appropriate information sheets together with consent forms. Written consent and assent will be required from both parent/guardian and child respectively for participation in the study. A favourable opinion was provided by the Dyfed Powys Research Ethics Committee for this project.

### Data Analysis

A quasi-experimental design will be used to compare differences in measured outcomes between pupils assigned to an intervention or control group. Initially, baseline data will be used to perform pearson's product moment correlation coefficients, followed by multiple regression analysis to estimate the significance of selected predictors on CVD risk factors. Dependant t-tests will be employed to assess differences between measures taken at baseline and post-intervention with independent t-tests utilised to detect differences in variables between groups. Statistical significance will be set at *P *≤ 0.05. Power calculations revealed that 100 participants from the exercise group and 60 participants from the control were required to achieve 80% power.

### Intervention

In addition to regular PE lessons (60 min duration), this intervention will aim to increase PA by an additional two hours per week, for 18 weeks. A cross-curricular PA intervention involving all academic subjects termed the 'Activity Knowledge Circuit' will be followed by year eight pupils. These participants will complete 3,200 m of brisk walking during a 60 mins subject (normally classroom based) lesson, where a metronome set at 130 beats per minute will provide a walking speed. Initial pilot work carried out suggests that walking at this intensity for 3,200 m elicits relative oxygen uptake values of 17.0 ± 1.3 ml^-1^kg^-1^min^-1^. Following 18-weeks, performing two intervention lessons per week, it is calculated that energy expenditure will equate to 4039 ± 1387 kcal, leading to a predicted weight loss of 1.2 ± 0.4 lbs, without accounting for confounding variables.

Each intervention lesson will be supervised by a subject teacher, and researcher or member of the PE department to ensure participants walk at the correct speed. Each participant will perform two intervention lessons per week, in addition to two regular PE lessons, thereby increasing activity by two hours a week. Intervention lessons will be integrated into the regular school timetable, and no more than four intervention lessons will be delivered by each curriculum subject during the 18-week period. Short tasks, in line with current curriculum, will be provided by subject teachers for participants to complete at stations set every 400 or 800 m. Participants will therefore, still follow national curriculum whilst exercising at the same time. Tasks are to be designed by teachers to last no longer than 60 seconds at each station. Physical Education lessons are to be implemented as usual.

Normally, all intervention classes will be performed outside on school premises on a pre-determined course. In adverse weather conditions, a course of equal distance has been arranged undercover, again on school premises, so preventing any disruption to the intervention schedule. Participants will wear school uniform whilst taking part, but suitable footwear will be advised. Registers are to be kept during each intervention-led lesson to monitor absentees.

### Measurements

**Figure 1:**
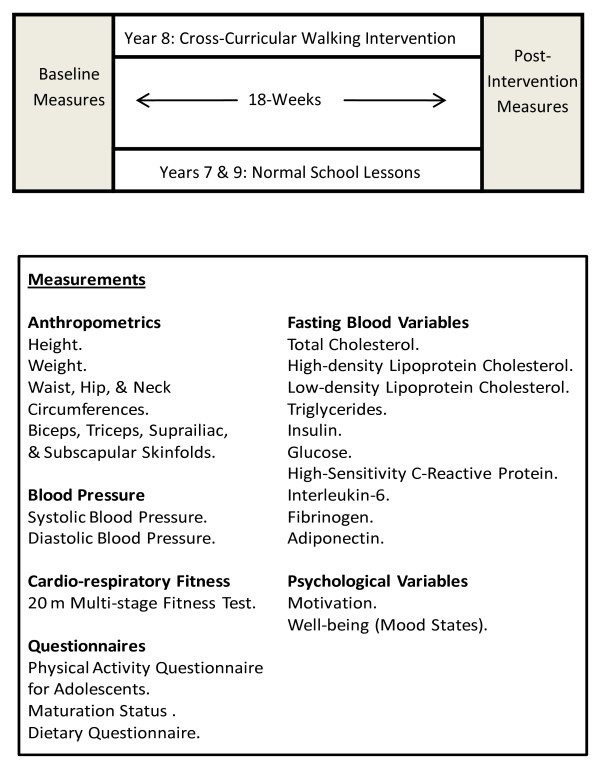
**Study design including measurements**.

All measurements will be performed pre- and post-intervention for both intervention and control groups (Figure 1). These are to be carried out by trained, gender specific individuals during PE lessons and on school premises to ensure minimal disruption to the curriculum. Individuals performing measurements will be CRB checked prior to data collection. Pre- and post-intervention measurements will be voluntary and last no longer than three weeks. Participants will be informed that they may withdraw at any point and that all information collected would be confidential.

#### Anthropometrics

Stature will be measured to the nearest millimetre using a portable stadiometer (Seca Stadiometer, Cardiokinetics Ltd. Salford, UK). Body mass will be measured to the nearest tenth of a kilogram using balance beam scales (Seca 710 Health Scales, Cardiokinetics Ltd. Salford, UK). Body mass index may then be calculated as body mass divided by stature squared and expressed as kilograms per metre squared. Waist and hip circumferences are to be measured whilst in a standing position using anthropometric tape. Waist circumference will be obtained laterally, midway between the lowest point of the rib cage and iliac crest, and measured at the end of a gentle expiration. Hip circumference will be taken at the widest point over the buttocks. Skinfold measurements obtained by callipers (Harpenden Skinfold Callipers, British Indicators, West Sussex, UK), will be measured at the biceps, triceps, subscapular, and suprailiac sites according to standard techniques [36]. A third skinfold measurement will be taken if the first two measurements differ by more than 1.0 mm. Summation of all four skinfolds will provide total skinfold thickness (TSF).

#### Blood Pressure

Blood pressure measurements will be performed using an automated blood pressure monitor (Dinamap XL Automatic Blood Pressure Monitor, Critikron Inc., Tampa, FL) following ten minutes of participants sitting quietly. The cuff will be positioned on the upper left arm and three measurements will be obtained with an average of the second and third used for data analysis [37]. Measures of systolic and diastolic blood pressure are to be recorded.

#### Aerobic Fitness Profile

Physical fitness will be estimated indirectly according to the participants' performance on the 20 m shuttle run (20MST) [38]. Each stage of the test is made up of several shuttle runs and the numbers of shuttles performed will be recorded. Higher shuttle scores will indicate higher levels of cardiovascular function. Testing will take place in the school's sports hall and consistent verbal encouragement will be given throughout. All participants will complete a prescribed warm up and cool down of light jogging and stretching. This test has previously been shown to be a valid assessment of maximal oxygen uptake in young individuals [39].

#### Questionnaires

The physical activity questionnaires for adolescents (PAQ-A) is to be used to report physical activity behaviour. This has previously been reported to be a valid and reliable measure of PA [40]. Each participant will record the amount of PA performed during the previous week by using a scale of 1-5; whereby 5 indicates high activity patterns and 1 represents low activity. Self-reported maturation status will be determined via questionnaire [41]. Participants will use recall to indicate their current stage of development. Self reported diet questionnaires will be provided for participants to complete at home. This includes a seven day food diary where participants record exactly what is consumed for breakfast, lunch, and dinner, as well as any snacks and drinks between meals. The diaries will be analysed for average daily kilojoules, percentage of total fat, saturated fat, carbohydrate, protein, and fibre by Health Options Ltd. (Health Options Ltd, Eastbourne, East Sussex, UK).

#### Blood Sampling and Analysis

Following an overnight fast, venous blood samples will be collected between 9.00 and 10.00 a.m. the following day. Participants will be required to assume a seated position for 30 minutes prior to sampling to control for plasma volume shifts [42]. Blood samples will be collected by qualified phlebotomists, and a health professional will be in attendance throughout. Breakfast will be provided to all participants immediately following sampling in the school canteen. Within two hours, all blood samples are to be transported to the laboratory and analysed immediately. Samples will be analysed for total cholesterol (TC), low-density lipoprotein cholesterol (LDL-C), HDL-C, TG, insulin, glucose, high-sensitivity C-reactive protein (hs-CRP), interleukin-6 (IL-6), adiponectin (high molecular weight), and fibrinogen (Fg).

### Psychological Measures

A number of psychosocial variables have been found to influence motivation to engage in PA, for example, readiness to change; self-efficacy and beliefs in ability; self-perception; goal orientation; enjoyment of activity. The aim of measuring motivation is to help develop strategies to increase the effectiveness of the intervention. One of the most enduring theoretical approaches to the study of motivation for PA is Self-Determination theory [43]. The measures selected are underpinned by this theoretical approach, and have all been well validated for use with the proposed sample:

Physical activity has been shown to reduce levels of anxiety and depression in the general population. Symptoms of anxiety and depression will be measured in the proposed sample by using well validated standardised instruments as detailed below:

#### Motivation

Readiness for change will be measured using five questions based on Kearney et al. [44]. Behavioural regulation in PA will be measured using the perceived locus of causality questionnaire (PLOC) [45]. The Task and Ego Orientation in Sport Questionnaire (TEOSQ) will be used to measure achievement orientation [46]. The Self Perception Profile for Adolescents will assess perceptions of competence including global self worth as well as perceptions of scholastic and athletic competence, physical appearance, social acceptance and behavioural conduct [47]. Beliefs in ability (self-efficacy) will be measured using the Conceptions of the Nature of Athletic Ability Questionnaire (CNAAQ-2) [48]. Enjoyment of PA will be measured using the Physical Activity Enjoyment Scale (PACES) [49]. These measures will be taken pre and post intervention to assess causal and moderating effects on outcomes.

#### Psychological Well-being (Mood States)

Anxiety will be measured using the State/Trait Anxiety Inventory for Children (STAI-CH) [50]. Depression will be measured using the Centre for Epidemiological Studies Depression Scale for Children (CES-DC) [51]. The d2 test of attention will be used to measure selective attention and mental concentration [52].

### Feedback

Feedback to both participants and parents/guardians will be provided following completion of post-intervention measurements. Providing feedback prior to the intervention may lead to alterations in lifestyles leading to a confounding effect. Feedback is to be provided on an individual and group basis. Individuals will be provided with pre- and post-intervention results, together with an explanation of results accompanied by suitable advice to modify lifestyle. Group feedback will be provided to the schools headmaster, and any abnormal findings are to be reported to participants' general practitioner.

## Discussion

Previous school-based PA interventions resulting in modest improvements to CVD risk status have typically been improvements to current PE curriculum or the introduction of extra-curricular sessions [15,30,31]. Our intervention will increase PA by an additional two hours per week by introducing brisk walking into curriculum subjects. This may be a cost effective strategy to increase PA in schools that could be implemented long term, targeting all children. By employing a cross-curricular intervention, PA becomes part of participants' school routine, therefore reducing the option to withdraw unless injury or illness prevents them from taking part.

Although most school-based PA intervention studies have focused on improving obesity, positive responses to other cardiovascular risk factors have been reported. Significantly lower systolic and diastolic blood pressures were reported by Bayne-Smith et al. [28], however, only girls were studied and the intervention included lectures on nutrition and behaviour modification as well as vigorous PA. Positive responses to BMI and serum lipid levels were reported by Manios et al. [27] by introducing an intervention to improve both diet and PA. Our study design will highlight the effect of introducing two hours of brisk walking a week on traditional CVD risk factors including obesity, blood lipid and lipoprotein profile and blood pressure. Evidence will also be provided on the effect of walking on other CVD risk factors including hs-CRP, IL-6, adiponectin, and Fg. Little information exists with regards to the effects of school-based exercise interventions on such variables.

The inclusion of motivational and psychological variables addresses a gap in research of this nature. It is clear that psychosocial factors are important in understanding the reason why young people engage (or fail to engage) with exercise programs. Furthermore, the psychological outcomes of participation (possible improvements in mood and concentration) and the extent to which they act as motivators for participation should be considered. If it can be established that increased participation in exercise has a notable effect on motivation to participate in exercise and psychological well-being together with improvements in CVD risk status, this may lead to long-term implications such as improved peer relations and school performance.

One of the main limitations to this study includes use of non-randomised sampling, whereby participants from year eight are to be selected to participate in the intervention, with participants from both years seven and nine making up the control group. This is unavoidable due to the manner in which the intervention is to be implemented. Randomly selecting participants from years seven, eight, and nine would lead to children of the same class being divided into intervention and control groups. This would cause too great a disruption to the school's regular curriculum, as well as requiring additional teachers. We considered assigning a whole year group to the intervention, and including maturation matched participants from other year groups as controls, is deemed more practicable. Future studies may look to involve a greater number of schools, allowing the opportunity to randomly assign different schools to an intervention or control group.

## Competing interests

The authors declare that they have no competing interests.

## Authors' contributions

NT, GK, SF and JR designed and wrote the original proposal. This has been modified and adapted by JB, BD, AR and KM. All authors have read and approved the final manuscript.

## Pre-publication history

The pre-publication history for this paper can be accessed here:

http://www.biomedcentral.com/1471-2458/9/466/prepub
